# Asymptomatic hepatic portal venous gas with gastric emphysema as a chronic complication of gastrostomy tube placement: a case report

**DOI:** 10.1186/s13256-016-1037-x

**Published:** 2016-08-24

**Authors:** Toyoaki Sawano, Tsuyoshi Nemoto, Masaharu Tsubokura, Claire Leppold, Akihiko Ozaki, Shigeaki Kato, Yukio Kanazawa

**Affiliations:** 1 Department of Surgery, Minamisoma Municipal General Hospital, 54-6 Takamicho 2 chome, Haramachi, Minamisoma, Fukushima 975-0033 Japan; 2 Department of Home Medical Care, Minamisoma Municipal General Hospital, Fukushima, 975-0033 Japan; 3 Division of Social Communication System for Advanced Clinical Research, Institute of Medical Science, University of Tokyo, Tokyo, 108-0071 Japan; 4 Department of Research, Minamisoma Municipal General Hospital, Fukushima, 975-0033 Japan; 5 Department of Internal Medicine, Soma Central Hospital, Fukushima, 976-0016 Japan; 6 Department of Gastroenterology, Minamisoma Municipal General Hospital, Fukushima, 975-0033 Japan

**Keywords:** Percutaneous endoscopic gastrostomy, Complication, Gastric emphysema, Emphysematous gastritis, Stroke

## Abstract

**Background:**

Percutaneous endoscopic gastrostomy feeding is widely used as a route for enteral feeding for patients with impaired swallowing ability, particularly in older patients. Hepatic portal venous gas is a condition that may arise from several causes. Hepatic portal venous gas that develops after an endoscopic procedure is generally reported to be nonfatal, yet there is little information available concerning the characteristics of hepatic portal venous gas as a chronic complication of percutaneous endoscopic gastrostomy feeding.

**Case presentation:**

We experienced a case of hepatic portal venous gas that happened to be detected in an 81-year-old Japanese man with long-term percutaneous endoscopic gastrostomy use who was admitted to our hospital with aspiration pneumonia. While aspiration pneumonia was treated with antibiotics and suspension of tube feedings, he recovered from hepatic portal venous gas without any treatment.

**Conclusions:**

The presence of a percutaneous endoscopic gastrostomy tube may have induced hepatic portal venous gas through a mechanism in which vomiting led to increased abdominal pressure and eventually gastric emphysema. This case suggests that hepatic portal venous gas without any signs of bowel ischemia or emphysematous gastritis can resolve without treatment, which is a finding that could be helpful for clinicians who deal with those supported by percutaneous endoscopic gastrostomy feeding.

## Background

Percutaneous endoscopic gastrostomy (PEG) feeding is widely used as a route for enteral feeding, hydration, and medication administration for patients with impaired swallowing ability, particularly in older patients [1]. Although PEG feeding provides the safest and most effective way to maintain a patient’s nutritional state and preserve long-term health [1], it holds the potential for complications. Two common types of complications are those that occur immediately after the PEG insertion procedure, and those that develop over a longer period. For instance, pneumoperitoneum [2], ileus [3], esophageal and gastric perforation [4], and hepatic portal venous gas (HPVG) [5, 6] are reported as some of the complications that may occur immediately following PEG insertion. On the other hand, deterioration of the gastrostomy site [7], buried bumper syndrome [8], colocutaneous fistula [9], and complications related to tube feeding are classified as chronic complications [7].

HPVG may arise from several predisposing conditions such as bowel ischemia, elevation of digestive tract pressure, abdominal infection, or endoscopic procedures, among others [10, 11]. The mortality rate for HPVG with necrotizing colitis is approximately 75 % [11, 12], whereas the mortality rate for HPVG without necrotizing colitis is not as high, highlighting the importance of distinguishing whether cases of HPVG may be fatal or nonfatal particularly by ruling out necrotizing colitis [13]. In terms of HPVG as a PEG-related complication, only cases of HPVG occurring immediately following PEG insertion have been reported to date [5, 6]. HPVG after an endoscopic procedure is generally thought of as a benign complication [11], yet there is little information available concerning the characteristics of HPVG as a chronic complication of PEG feeding.

We present here a case of HPVG that happened to be detected in a patient with a past history of stroke and enteral feeding via PEG; the PEG had been inserted more than 6 months ago. Given the fact that large numbers of older patients are supported by PEG, it is likely that clinicians may experience HPVG in patients with PEG tubes. The present case report highlights that HPVG occurrence may be an important point of consideration for those maintaining the health of patients with chronic PEG feeding.

## Case presentation

An 81-year-old Japanese man with a past history of stroke, hypertension, and dyslipidemia presented to our hospital because of respiratory failure. He had presented to the department of neurosurgery in our hospital 9 months earlier with a diagnosis of right cerebral infarction. He then developed left motor hemiparesis, left sensory deficit, and aphasia. He was operated on for PEG tube replacement (Button-type gastrostomy tube; Olympus Medical System Co., Ideal PEG Kit) by a gastroenterologist without complications 6 months before admission. Soon after the procedure he developed recurrent cerebral infarction and it became difficult to communicate with him. He had presented with aspiration pneumonia 3 weeks before the present admission. After being treated with antibiotic therapy for 2 weeks, he was discharged home 3 days prior to the present admission.

**Fig. 1:**
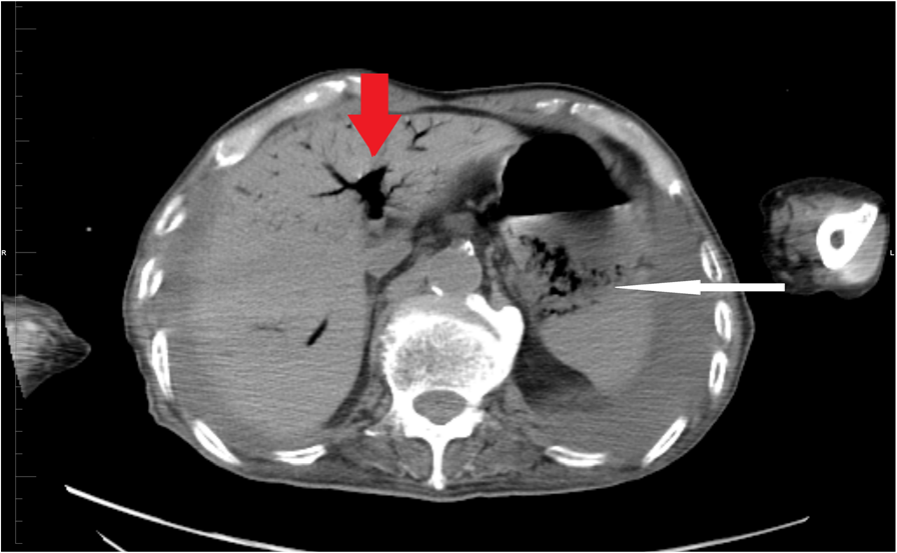
Computed tomography obtained on admission day showed hepatic portal venous gas (*thick arrow*) and air within the gastric wall (*thin arrow*)

**Fig. 2:**
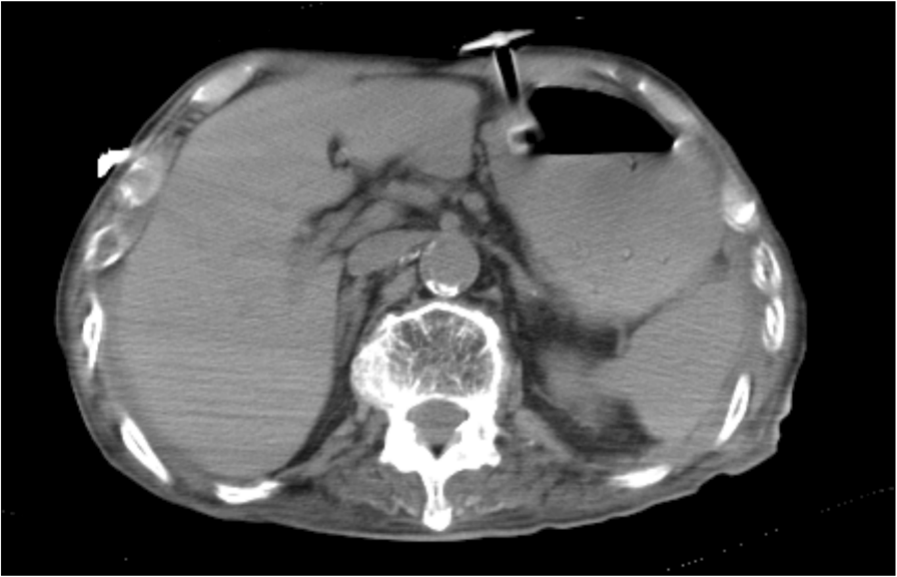
Computed tomography obtained 3 weeks before admission with diagnosis of aspiration pneumonia showed no hepatic portal venous gas and no gas within the wall of stomach in the visualized portions of the upper abdomen

**Fig. 3:**
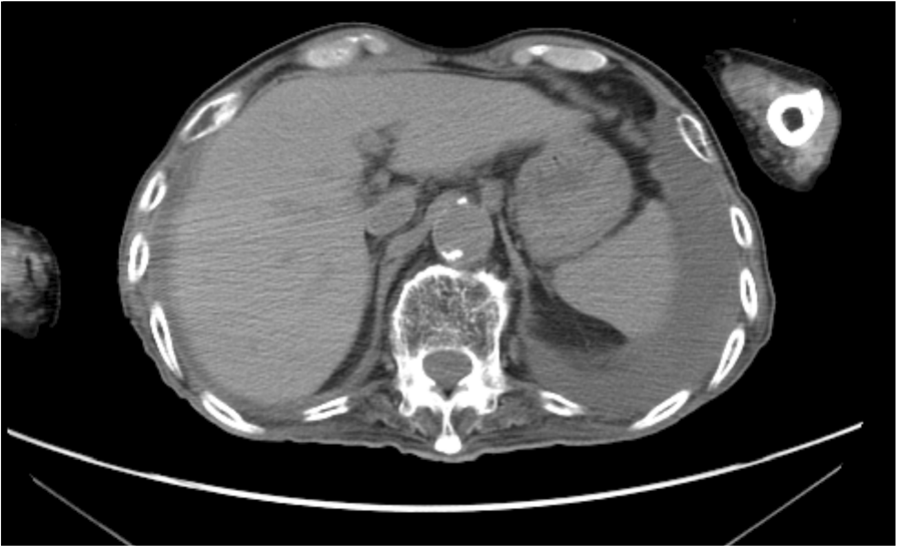
Follow-up computed tomography on hospital day 14 showed resolution of hepatic portal venous gas and air within the stomach wall

On admission day of the present case, he had developed diarrhea and begun vomiting in the morning. After vomiting, he had been well until the evening; however, he then went into respiratory failure. His family called emergency medical services (EMS), and he was transported to our hospital. On examination in our emergency room, his temperature was 38.6 °C, blood pressure 155/70 mmHg, his pulse was 125 beats per minute (bpm), respiratory rate 22 breaths per minute, with oxygen saturation of 94 % on 10 liters of ambient air per minute given by mask. On physical examination, crackles and wheezing were heard on bilateral lower lung fields, and there was no tenderness and peritoneal signs in all quadrants of his abdomen. Results of a laboratory examination showed pH 7.445, partial pressure of oxygen 82.9 mmHg, partial pressure of carbon dioxide 37.5 mmHg, base excess 1.9 mmol/L, white cell count 16,830/μl, C-reactive protein 4.30 mg/dl, aspartate transaminase 29 IU/l, alanine transaminase 27 IU/l, alkaline phosphatase 271 IU/l, lactate dehydrogenase 224 IU/l, urea nitrogen 22.1 mg/dl, and creatinine 0.62 mg/dL. Computed tomography (CT) showed HPVG (diagnosed by appearance of branching lucencies within 2 cm of liver capsule [11]) and gas within the wall of his stomach (Fig. 1). A CT obtained 3 weeks before admission with diagnosis of aspiration pneumonia showed no HPVG and no gas within the wall of his stomach in the visualized portions of his upper abdomen (Fig. 2). He received a 2-week course of antibiotic therapy (ampicillin-sulbactam) for aspiration pneumonia, and feeding products were changed to another type. Follow-up CT on hospital day 14 showed HPVG and air within his stomach wall diminishing (Fig. 3). His respiratory condition gradually improved. He was discharged on hospital day 17.

## Discussion

In the present case, HPVG arose in a patient supported by a PEG which had been inserted more than 6 months ago; his HPVG resolved without treatment.

The presence of a PEG tube may have induced HPVG through an increase of abdominal pressure due to vomiting. HPVG has several predisposing conditions, which include bowel ischemia, elevation of digestive tract pressure, abdominal infection, endoscopic procedure, gastric emphysema, and emphysematous gastritis [10, 11, 14]. In the present case, accumulations of gas within our patient’s stomach wall were found through a CT scan, leading to a diagnosis of gastric emphysema. Although it is impossible to confirm whether the presence of gastric emphysema in the present case was associated with HPVG, gastric emphysema is generally triggered by secondary mechanical injury of the stomach surface [15]. In addition, increased abdominal pressure due to vomiting before admission in the present case could have led to gastric emphysema via the PEG hole, although possible associations between gastric emphysema and PEG tubes have not yet been explored in the existing literature. While we cannot rule out other potential causes of HPVG, the above hypothesis is consistent with the clinical course of this case, and thus we find a high possibility for HPVG to have been associated with the PEG tube.

It appears that HPVG without any signs of bowel ischemia or emphysematous gastritis can resolve without any treatment in patients who are supported by chronic PEG feeding. HPVG was recognized to be fatal in the 1980s, yet recent increases in cases have demonstrated that non-symptomatic HPVG is not necessarily fatal [13]. In the present case, there were no acute abdominal symptoms on physical examination, and a blood test showed pH 7.445, base excess 1.9 mmol/L, and lactate dehydrogenase 224 IU/l. In addition, CT did not indicate pneumatosis intestinalis or obvious digestive wall thickening. It was therefore judged that there were no signs suggesting bowel ischemia or emphysematous gastritis. While aspiration pneumonia was treated with antibiotics as well as suspension of tube feedings, specific interventions such as gastric decompression or gastrectomy were not carried out. PEG feeding was re-started 7 days after admission, and HPVG was found to be diminishing without sequelae in a CT scan taken 14 days after admission.

In recognition of the growing numbers of older patients supported by PEG, the Japanese government has enforced a new law to reduce the medical fees for PEG placement [16]. There are many patients with long-term PEG use, particularly among older patients, and chronic complications such as HPVG seen in this case may be experienced by clinicians caring for these patients. Excluding patients with a need for acute surgery, it may be possible for non-fatal HPVG to be resolved without aggressive treatment, as seen in the present case.

## Conclusions

We experienced a case of HPVG that happened to be detected in a patient with a past history of stroke and enteral feeding by PEG; the PEG had been inserted 6 months earlier. HPVG may be associated with use of PEG tubes, but can be resolved without any treatment in patients without symptoms of bowel ischemia or emphysematous gastritis.

## Abbreviations

CT: Computed tomography
HPVG: Hepatic portal venous gas
PEG: Percutaneous endoscopic gastrostomy

## References

[1] Friginal-Ruiz AB, Lucendo AJ. Percutaneous Endoscopic Gastrostomy: A Practical Overview on its Indications, Placement Conditions, Management, and Nursing Care. Gastroenterol Nurs. 2015;38(5):354–66. 10.1097/SGA.0000000000000150 26422269

[2] Wiesen AJ, Sideridis K, Fernandes A, Hines J, Indaram A, Weinstein L, Davidoff S, Bank S. True incidence and clinical significance of pneumoperitoneum after PEG placement: a prospective study. Gastrointest Endosc. 2006;64(6):886–9. 10.1016/j.gie.2006.06.088 17140892

[3] Dulabon GR, Abrams JE, Rutherford EJ. The incidence and significance of free air after percutaneous endoscopic gastrostomy. Am Surg. 2002;68(6):590–3. 12079145

[4] Chirica M, Champault A, Dray X, Sulpice L, Munoz-Bongrand N, Sarfati E, Cattan P. Esophageal perforations. J Visc Surg. 2010;147(3):e117–28. 10.1016/j.jviscsurg.2010.08.003 20833121

[5] Bobba RK, Arsura EL. Hepatic portal and mesenteric vein gas as a late complication of percutaneous endoscopic gastrostomy tube placement in an elderly patient. Dig Dis Sci. 2005;50(2):411–4. 10.1007/s10620-005-1620-2 15745110

[6] Kadomatsu Y, Kojima T, Kohara M, Inamori M. Hepatic portal venous gas development following percutaneous endoscopic gastrostomy. Intern Med. 2013;52(1):153. 10.2169/internalmedicine.52.8782 23291693

[7] Blomberg J, Lagergren J, Martin L, Mattsson F, Lagergren P. Complications after percutaneous endoscopic gastrostomy in a prospective study. Scand J Gastroenterol. 2012;47(6):737–42. 10.3109/00365521.2012.654404 22471958

[8] Klein S, Heare BR, Soloway RD. The “buried bumper syndrome”: a complication of percutaneous endoscopic gastrostomy. Am J Gastroenterol. 1990;85(4):448–51. 2109527

[9] Saltzberg DM, Anand K, Juvan P, Joffe I. Colocutaneous fistula: an unusual complication of percutaneous endoscopic gastrostomy. JPEN J Parenter Enteral Nutr. 1987;11(1):86–7. 10.1177/014860718701100186 2950249

[10] Liebman PR, Patten MT, Manny J, Benfield JR, Hechtman HB. Hepatic–portal venous gas in adults: etiology, pathophysiology and clinical significance. Ann Surg. 1978;187(3):281–7. 10.1097/00000658-197803000-00012 PMC1396434 637584

[11] Kinoshita H, Shinozaki M, Tanimura H, Umemoto Y, Sakaguchi S, Takifuji K, Kawasaki S, Hayashi H, Yamaue H. Clinical features and management of hepatic portal venous gas: four case reports and cumulative review of the literature. Arch Surg. 2001;136(12):1410–4. 10.1001/archsurg.136.12.1410 11735870

[12] Seak CJ, Hsu KH, Wong YC, Ng CJ, Yen DH, Seak JC, Seak CK. The prognostic factors of adult patients with hepatic portal venous gas in the ED. Am J Emerg Med. 2014;32(9):972–5. 10.1016/j.ajem.2014.05.016 25043627

[13] Nelson AL, Millington TM, Sahani D, Chung RT, Bauer C, Hertl M, Warshaw AL, Conrad C. Hepatic portal venous gas: the ABCs of management. Arch Surg. 2009;144(6):575–81. discussion 581. 10.1001/archsurg.2009.88 19528392

[14] Matsushima K, Won EJ, Tangel MR, Enomoto LM, Avella DM, Soybel DI. Emphysematous gastritis and gastric emphysema: similar radiographic findings, distinct clinical entities. World J Surg. 2015;39(4):1008–17. 10.1007/s00268-014-2882-7 25446476

[15] Misro A, Sheth H. Diagnostic dilemma of gastric intramural air. Ann R Coll Surg Engl. 2014;96(7):e11–3. 10.1308/003588414X13946184901128 PMC4473470 25245715

[16] Revision of medical fee in 2014. In. Tokyo: Ministry of Health, Labour and Welfare; 2014.

